# Notes of an Interview with Brown, the “Mind-Reader”

**Published:** 1873-09

**Authors:** Henry M. Lyman

**Affiliations:** Prof. of Chemistry, Rush Medical College, Chicago; No. 533 West Adams St.


ARTICLE VII.—*Notes of an Interview with Brown, the "Mind-Reader."* By HENRY M. LYMAN, M.D., Prof. of Chemistry, Rush Medical College, Chicago.*

At 5 o'clock P. M., August 13, 1873, a small party of physicians, clergymen, and gentlemen interested in scientific studies, were assembled by invitation of the pastor, Rev. C. D. Helmer, in the parlors of the Union Park Church in this city, to meet the young gentleman whose peculiar endowments are attracting considerable attention at the present time. He soon appeared, with his mentor, Mr. Kelley, and proceeded at once to business.

Mr. Brown is a young man, twenty-one years of age; about five feet eight inches tall; would weigh not far from one hundred and twenty pounds; has dark hair, eyes and complexion; has a frank, open countenance; his manner is quick and alert; but there is about him nothing which would suggest the possession of any unusual endowment. His education has been that of a country boy in the public schools, and his occupation is that of a machin-

* Received too late for insertion in its proper place.

ist. His companion, Mr. Kelley, an elderly gentleman, well and favorably known by some of the residents of this city, stated that from infancy Mr. Brown's mother considered him a strange child, rather peculiar in his ways; but it was only eight years ago that his singular nervous susceptibilities were discovered. At that time, while wrestling one day with another boy, his opponent happened to brush the back of his left hand across his forehead. Instantly flashes of light seemed to radiate from his head. A repetition of the conditions was followed in every instance by the same result. This arrested his attention, and led him to experiment in various ways relative to the effects of physical contact with other persons, until he arrived at the discovery that if, when his eyes were closed, the back of the left hand of another were pressed against his forehead, his movements were subjected to the guidance of that person's will as long as such contact was maintained. To secure this subordination, the left hand must be used; and the limb must be a perfect member: arms that had ever been broken or seriously injured would not answer the purpose. Nor could this peculiar guidance be exercised by any one at all under the influence of liquor.

Having thus introduced his protege, Mr. Kelley stated that if any one of the party would conceal something, anywhere he pleased, Mr. Brown would conduct him to the spot. I therefore placed my lancet-case upon the projection of the middle hinge of a door on the right side of the lobby of the church, and returning to the parlor I determined to fix my thoughts, while being led by Mr. B., in the first place upon the white door-knob of the parlor door, then upon the knob of an opposite door, then upon the left-hand extremity of the lowest step of the stairs on the left side of the lobby, and finally upon the location of the lancet-case. I then gave my left hand to Mr. Brown who had been carefully blindfolded. Requesting me to keep my arm fully extended, and grasping my left hand with his left hand, he placed the palm of his right hand for nearly a minute upon my forehead. At the first instant of contact his muscles were tranquil, but almost immediately his hand began to tremble, and to tremble more violently the longer the contact was maintained. Having thus touched my forehead, he placed the palm of his right hand upon his own forehead for about ten seconds, and then quickly applied the back of my left hand to the same place. He at once began to move; and concentrating my thoughts as rapidly as possible upon the path which I had previously marked out in my own mind, I found myself speedily dragged along to the parlor door, where Mr. B. bowed his head to the white door-knob. Then raising himself he began to move towards the other door before I fairly knew what he was about. After taking two or three steps in that direction he seemed to waver, and being now recovered from the mental confusion incident upon surprise, I concentrated my thoughts upon the stairs, and was immediately led to the left-hand extremity of the lowest step of the stair in the lobby. Here he stopped, and seeming inclined to bend his body downwards, I mentally addressed him the words, "Down! down! down there!" He immediately bowed his head till the forehead almost touched the step, and said, "It is there;" at the same time letting go my hand. We had not traversed the last stage of the route which I had planned, but it was evident that as long as physical contact was preserved, the direction of the course of his movements was largely under the control of my own will.

Returning to the parlor from which we had started, Dr. I. N. Danforth concealed his knife in a hymn-book which was placed upon a pile of books in an adjoining room, and then gave his hand to Mr. Brown. After perfecting the contact by the usual manipulations, he started with the doctor upon a long and devious chase all around the interior of the building, but finally led him to the book which contained his knife. Dr. D. is a small man with sandy hair, a slender frame, and a nervous-sanguine temperament; and having failed in his intention to direct Mr. Brown by the *shortest possible course* to his knife, we decided to repeat this experiment with a person of different temperament,—Dr. C. W. Earle, a large and powerful man, with dark hair and complexion.

Dr. Earle suspended his watch in a closet, and then surrendered his hand to Mr. B. who led him a wilder goose chase than he had led Dr. Danforth. After dragging him up stairs and all over the audience-room of the church, Mr. Brown gave up the search, saying that he must rest. He stated that if overheated his power was greatly diminished, and that it was greater in cool weather than in hot. While resting we ascertained that his temperature was $98\frac{1}{2}°$ F.; pulse normal. Temperature of the atmosphere $75°$

F. Dr. Earle stated that during the experiment he had concentrated his thoughts upon the watch itself rather thán upon the place of its concealment. Cautioning him to converge his attention upon the locality, Mr. Brown resumed the experiment, and immediately dragged the doctor at top speed to the place where his watch was hanging. During the time of his rest he had no communication with any one who knew where the article had been hidden.

The rapidity and certainty of his movements during these experiments were remarkable. His gait was almost a run; and he steered clear of every obstacle with the precision of ordinary eyesight. This, so long as his companion used his own eyes. We therefore proceeded to vary the experiment. Having arranged the blinder, one of our number placed a small watch-key upon the centre of the mat before the parlor door. Then placing himself in communication with Mr. Brown, he kept his own eyes continually closed. It was evident that there was no guiding impulse in action. The pair stumbled aimlessly around the room, over the chairs and against the partitions,—the blind leading the blind,—until Mr. B. exclaimed, "There is something very strange about this gentleman; I think you must be experimenting with me!" He then stopped, and removed the handkerchief from his eyes; but being assured that all was right, he renewed the search. His companion now kept his eyes open, and was soon taken to the place where he had deposited the key. He stated that while his eyes were closed he lost all idea of direction, and could not tell in what part of the room he was; but when his eyes were open, as soon as he addressed Mr. Brown, mentally, with the words "Go there, go there!" he found himself instantly obeyed.

Another experiment proved that the volitional impulse might be conveyed through the medium of a compound conductor. Let A represent Mr. Brown; let C represent the individual by whom he is to be guided; and let B represent a third person. Now if C with his left hand grasps the naked left arm of B, while the back of the left hand of B is applied to the forehead of A, he can guide the movements of A, even though both A and B are blindfolded and previously ignorant of the place to which C would direct A. This was demonstrated to our complete satisfaction.

The last experiment of the series was the following: Placing myself in communication with Mr. Brown, I requested him to point in the direction of the object upon which my thoughts were concentrated. I had fixed upon the top of the church-spire for the object; but no sooner had we commenced the experiment than I became aware of a ludicrous uncertainty regarding the proper angle of elevation of the line which should lead my thoughts to the top of the spire. Mr. B. was whirling me rapidly round and round, so I hastily abandoned the attempt to soar so high, and endeavored to concentrate my will upon an imaginary line up and down the centre of the eastern *facade* of the church. He at once ceased to turn around, and after wavering a few seconds he pointed, not exactly east, towards the centre of the *facade*, but east-north-east, towards the centre of the tower which supports the spire upon which my thoughts had been originally directed.

In answer to our inquiries, Mr. Brown stated that when placed in communication with another person, as previously described, his movements are guided by a light which seems to stream from his head in the direction of the object which he seeks. This light appears to him very much like a gas-light viewed through smoked glass. At the commencement of an experiment, or if it does not proceed in a satisfactory manner, the light seems broken up into numerous smaller lights which flash distractingly in every direction; but soon they all concentrate into a single flame which seems to dart in the direction which he must follow. Sometimes the light beams without deviation from the right course; but often it describes a zig-zag path for his guidance.

He stated that his health was perfect, and that he seldom felt fatigued by his exhibitions, though it is necessary for him to rest often between the experiments in order to maintain the acuteness of his faculty. Sometimes, however, experiments with certain individuals are very exhausting, as, for instance, his experiment with Dr. Earle. The peculiarity of his case consists in the fact that his singular nervous susceptibility does not require the production of artificial somnambulism or *hypnotism* as the condition of its manifestation; nor does it at all interfere with the integrity of his will. It is not in obedience to an irresistible impulse that he pursues the luminous apparitions which seem to proceed from his head, but he can always follow or refrain from following their guidance, precisely as a person in the dark can follow or refrain from following the movements of a lantern which flickers along the path before him.

No. 533 West Adams St.

